# Draft Genome Sequences of the Obligatory Marine Myxobacterial Strains Enhygromyxa salina SWB005 and SWB007

**DOI:** 10.1128/genomeA.00324-18

**Published:** 2018-04-26

**Authors:** Jamshid Amiri Moghaddam, Anja Poehlein, Katja Fisch, Mohammad Alanjary, Rolf Daniel, Gabriele M. König, Till F. Schäberle

**Affiliations:** a Institute for Pharmaceutical Biology, University of Bonn, Bonn, Germany; b Department of Genomics and Applied Microbiology and Göttingen Genomics Laboratory, Georg-August-University Göttingen, Göttingen, Germany; c Institute for Insect Biotechnology, Justus Liebig University Giessen, Giessen, Germany; d Department of Microbiology and Biotechnology, University of Tübingen, Tübingen, Germany; e German Center for Infection Research (DZIF), Partner Site Cologne-Bonn, Bonn, Germany

## Abstract

The two marine myxobacterial strains Enhygromyxa salina SWB005 and SWB007 were isolated from coastal soil samples using Escherichia coli as bait for these predatory strains. These strains produce unique specialized metabolites. Genomes were assembled into 312 contigs for E. salina SWB005 (9.0 Mbp) and 192 contigs for E. salina SWB007 (10.6 Mbp).

## GENOME ANNOUNCEMENT

Enhygromyxa salina is a marine myxobacterium, and strains of this species have been isolated from different locations around the globe (1, 2). However, to date, only a few obligatory marine myxobacteria have been isolated, and only one genome was available (i.e., from the strain E. salina DSM 15201). Like other myxobacteria, these genomes are large, ranging from 8 to 10 Mbp, and harbor many putative biosynthetic gene clusters (BGCs) coding for the production of specialized metabolites. The strains analyzed here are producers of the natural products salimabromide, salimyxins, and enhygrolides (3–5). E. salina strains SWB005 and SWB007 were isolated from marine sediments from the coast of Santa Barbara, CA (SWB005), and Prerow, Germany (SWB007) (2, 3).

Genomic DNA of both E. salina strains were extracted from fruiting bodies, which appeared after several days of fermentation in artificial seawater (ASW) VY/4 liquid medium (6). DNA was isolated using the GenElute bacterial genomic DNA kit (Sigma-Aldrich). Extracted DNA was used to generate Illumina shotgun paired-end sequencing libraries, which were sequenced with a MiSeq instrument and the MiSeq reagent kit version 3, as recommended by the manufacturer (Illumina, San Diego, CA, USA). Quality filtering using Trimmomatic version 0.36 (7) resulted in 3,773,950 and 3,458,266 paired-end reads for E. salina strains SWB007 and SWB005, respectively.

Assembly resulted in 192 contigs (>500 bp) with an average coverage of 73-fold for E. salina SWB007 and 312 contigs (>500 bp) with an average coverage of 75-fold for E. salina SWB005. The assemblies were validated and the read coverage was determined with Qualimap version 2.1 (8). The resulting draft genomes are 10,602,813 bp (E. salina SWB007) and 9,010,436 bp (E. salina SWB005) in length, and the G+C contents are 68.1% and 69.5% (difference, 1.4%), respectively. Automatic annotation and identification of rRNA and tRNA genes were performed using the software tool Prokka (9). This yielded 2 rRNA genes, 78 tRNA genes, 3,682 protein-encoding genes with function prediction, and 3,265 genes coding for hypothetical proteins for strain SWB005 and 4 rRNA genes, 57 tRNA genes, 4,253 protein-encoding genes with function prediction, and 3,987 genes coding for hypothetical proteins for strain SWB007.

*In silico* DNA-DNA hybridization (isDDH) was performed based on the identities/high-scoring segment pairs (HSP) length formula (10) and produced a value of 26.10% (+23.8%, −28.6%), which failed the isDDH cutoff of ≥70% that would have determined them to be the same species. The average nucleotide identity (ANI) was calculated to be 78.7%. Therefore, the *in silico* parameters ANI, isDDH, and difference of the G+C values define these strains as two distinct species of the genus Enhygromyxa.

Using the antiSMASH version 4.0.0 tool (11) for the analysis of the genomes revealed 40 BGCs in E. salina SWB005 and 46 BGCs in E. salina SWB007, indicating the high potential of these bacteria for biosynthesis of specialized metabolites.

### Accession number(s).

This whole-genome shotgun project has been deposited at DDBJ/ENA/GenBank under the accession numbers PVNK00000000 (E. salina SWB005) and PVNL00000000 (E. salina SWB007). The versions described in this paper are versions PVNK01000000 and PVNL01000000, respectively.
